# Differential beta desynchronisation responses to dynamic emotional facial expressions are attenuated in higher trait anxiety and autism

**DOI:** 10.3758/s13415-022-01015-x

**Published:** 2022-06-27

**Authors:** Chengetai Alice Charidza, Helge Gillmeister

**Affiliations:** 1 Department of Psychology, University of Essex, Colchester, CO4 3SQ UK; 2 Department of Psychology, University of Exeter, Washington Singer Building, Perry Road, Exeter, EX4 4QG UK

**Keywords:** Emotional facial expressions, Mu, Alpha, Beta, Event-related desynchronisation, Anxiety, Autism

## Abstract

Daily life demands that we differentiate between a multitude of emotional facial expressions (EFEs). The mirror neuron system (MNS) is becoming increasingly implicated as a neural network involved with understanding emotional body expressions. However, the specificity of the MNS’s involvement in emotion recognition has remained largely unexplored. This study investigated whether six basic dynamic EFEs (anger, disgust, fear, happiness, sadness, and surprise) would be differentiated through event-related desynchronisation (ERD) of sensorimotor alpha and beta oscillatory activity, which indexes sensorimotor MNS activity. We found that beta ERD differentiated happy, fearful, and sad dynamic EFEs at the central region of interest, but not at occipital regions. Happy EFEs elicited significantly greater central beta ERD relative to fearful and sad EFEs within 800 - 2,000 ms after EFE onset. These differences were source-localised to the primary somatosensory cortex, which suggests they are likely to reflect differential sensorimotor simulation rather than differential attentional engagement. Furthermore, individuals with higher trait anxiety showed less beta ERD differentiation between happy and sad faces. Similarly, individuals with higher trait autism showed less beta ERD differentiation between happy and fearful faces. These findings suggest that the differential simulation of specific affective states is attenuated in individuals with higher trait anxiety and autism. In summary, the MNS appears to support the skills needed for emotion processing in daily life, which may be influenced by certain individual differences. This provides novel evidence for the notion that simulation-based emotional skills may underlie the emotional difficulties that accompany affective disorders, such as anxiety.

**Supplementary Information:**

The online version contains supplementary material available at 10.3758/s13415-022-01015-x.

## Introduction

We can find ourselves unexpectedly smiling upon seeing others’ positive emotional facial expressions (EFEs). This automatic tendency to mimic EFEs is thought to result from sensorimotor simulation within the brain (Adolphs et al., 2000; Bastiaansen et al., 2009; Kragel & LaBar, 2016; Wood et al., 2016). Emotion-based sensorimotor simulation theories propose that, as a result of not having access to other people’s emotional states, we translate visual motor cues (e.g., muscle movements, such as smiles) onto our own systems to recognise such states (Bastiaansen et al., 2009; Wicker et al., 2003; Wood et al., 2016). As such, sensorimotor simulation is thought to be an important factor in how we can accurately understand EFEs (Adolphs et al., 2000; Kragel & LaBar, 2016).

Sensorimotor simulation regions are part of the extended mirror neuron system (MNS; i.e., involving the somatosensory cortices and the insula; Bastiaansen et al., 2009; Kragel & LaBar, 2016; Pineda, 2008; Wood et al., 2016), which could be interconnected with the prefrontal cortex (PFC), limbic system, and the default mode network (Molnar-Szakacs & Uddin, 2013; Perry et al., 2017; Rizzo et al., 2018). Overall, this broadly distributed network is thought to underlie the mental representation of our own and others’ actions, sensations, and emotions (Bastiaansen et al., 2009; Pineda, 2008; Rizzo et al., 2018; Wood et al., 2016).

Although the MNS, and specifically its sensorimotor regions, are emerging as neural substrates involved in EFE recognition (Moore & Franz, 2017; Rymarczyk et al., 2019; Wood et al., 2016), it is not clear how specifically the MNS is involved in the delineation of the broad spectrum of EFEs. The differentiation of a range of EFEs (e.g., all six basic emotions suggested by Ekman & Friesen, 1971) within the MNS thus remains an important but relatively unexplored topic.

In electroencephalography (EEG) studies, sensorimotor MNS activity has been indexed by event-related desynchronisation (ERD) within mu frequencies. The mu (Rolandic) rhythm is comprised of alpha and beta frequencies over the sensorimotor regions (i.e., central/vertex electrodes), which reduce in power during the execution and observation of actions as a result of increased sensorimotor cortical activity (Fox et al., 2016; Hari et al., 1998; Pfurtscheller & Lopes da Silva, 1999). Mu desynchronisation in response to social stimuli (e.g., EFEs) is typically greater within right hemisphere electrodes, which has been demonstrated with infants, children, and adults (Arnett et al., 2019; Moore et al., 2012; Rayson et al., 2016). This right hemispheric dominance is in line with suggestions that right somatosensory cortical activity is particularly important for the recognition of facial and vocal emotional expressions (Adolphs et al., 2000; Kragel & LaBar, 2016).

It is plausible that sensorimotor MNS responses may aid in the delineation of emotions based on their discrete emotion value. Emotions motivate action readiness to promote survival in varying ways; for example, happiness tends to be related to more prosocial behaviours, whereas anger tends to be related to more antisocial behaviours (Aknin et al., 2015; Chow et al., 2008; Ekman, 1992; Keltner & Gross, 1999; Xu et al., 2015). As a result, mu suppression may evidence a unique signature for each emotion.

In line with this, Moore et al. (2012) showed that the observation of static EFEs of happiness and disgust evoked distinct temporal profiles of mu desynchronisation in right-lateralised central electrodes. At 500 ms post-stimuli, static disgusted faces elicited significantly greater alpha desynchronisation than static happy faces; then approximately 600 - 1,500 ms post-stimuli, happy faces elicited greater and longer lasting desynchronisation than disgust. The more rapid simulation of disgust may reflect an evolutionary mechanism necessary to avoid contact with disease or contaminated substances (Curtis et al., 2004; Oaten et al., 2009). The subsequent sustained sensorimotor simulation of happiness may indicate individuals’ motivation to simulate prosocial or positive faces relative to antisocial or negative faces, as prosocial behaviour provides benefits, such as a higher sociometric status (Cashdan, 1998; Hess & Fischer, 2014; Matsumoto & Kudoh, 1993; Nikitin & Freund, 2019; van Baaren et al., 2004).

Whilst past studies provide evidence that sensorimotor simulation may contribute to the recognition of distinct EFEs, several questions remain unanswered. First, do mu suppression differences between happiness and disgust reflect an emotional distinction based on the discrete value (e.g., basic emotions model; Ekman, 1999; Ekman et al., 1987), the action motivational value (approach vs. avoidance; e.g., left frontal hemispheric dominance for approach emotions and right frontal hemispheric dominance for withdrawal emotions; e.g., Coan et al., 2001; Davidson et al., 1990), or indeed a combination of the theories? Understanding the extent to which the MNS differentiates a broad range of EFEs (e.g., all six basic emotions) could be a first step towards answering this question.

Second, can alpha suppression profiles be unequivocally characterised as sensorimotor activation profiles when alpha is known to reflect a variety of cognitive processes, including visual attention and behavioural inhibition (Hobson & Bishop, 2017; Klimesch, 2012)? Mu suppression studies have tended to limit their analyses to central electrodes (Rayson et al., 2016) or did not explicitly state how emotions were differentiated within central sensorimotor regions (Moore & Franz, 2017). Limiting mu to central electrodes could hinder how we understand the MNS’s relation to wider networks (e.g., attentional networks; Perry & Bentin, 2010). For example, occipital alpha rhythms may reflect activity related to central sensorimotor MNS regions (Csukly et al., 2016) but also could reflect differential engagement of stimulus- or task-driven attentional processes (Capotosto et al., 2009; Hopfinger et al., 2000; Vanni et al., 1996) that are not directly related to the MNS (Hobson & Bishop, 2016, 2017). Moreover, mu suppression studies with EFEs have tended to focus on one frequency band (alpha; Moore et al., 2012), although it is known that alpha and beta show nuances in how they respond to sensory (tactile) versus motor components of facial and bodily actions (Angelini et al., 2018; Coll et al., 2017; Cooper et al., 2013; Ritter et al., 2009; Siqi-Liu et al., 2018). Alpha and beta ERD should therefore both be explored more broadly over the scalp.

Third, it is not clear how MNS emotion specificity may differ with maladaptive emotional processing at subclinical and clinical levels. Literature has linked atypical MNS activity with emotional processing difficulties in individuals with depression, schizophrenia, and autism spectrum conditions (ASCs; Canbeyli, 2010; Canbeyli, 2013; Cooper et al., 2013; Neumann et al., 2014; Siqi-Liu et al., 2018; Sperling et al., 1999; Yin et al., 2018), prompting the exploration into alternative therapies, such as neurofeedback training (NFT; Pineda et al., 2014) for MNS regions to improve emotional processing (Yuan & Hoff, 2008). However, at present there is little research investigating whether atypical MNS activity also may underlie individual differences in anxiety (Rachman, 2013; Steimer, 2002), which can be defined as a temporary affective state (i.e., state anxiety), but also as a stable and enduring affective state (i.e., trait anxiety) within the general population (Barlow et al., 2014; Duval et al., 2015; Gawda & Szepietowska, 2016; Kessler et al., 2005; Lengel et al., 2016; Rachman, 2013; Ree et al., 2008; Wang et al., 2019). Trait anxiety and anxiety disorders are relevant within the context of emotion processing and the MNS for three key reasons.

Firstly, anxiety disorders tend to be comorbid and have symptom overlap (e.g., emotion regulation difficulties) with emotion-related conditions linked to the MNS regions, such as depression (Goodwin, 2015; Hirschfeld, 2001; Wang et al., 2019), ASCs (Nadeau et al., 2011; Wood & Gadow, 2010), and schizophrenia (Buckley et al., 2009; Temmingh & Stein, 2015).

Secondly, it has been suggested that high levels of trait anxiety and anxiety disorders can influence how EFEs are perceived and interpreted (Dijk et al., 2018; Ewbank et al., 2009; Ferguson et al., 2021; Mennin et al., 2009; Yu et al., 2018). For example, more anxious individuals tended to perceive neutral faces as more threatening (Cooney et al., 2006; Peschard & Philippot, 2017).

Thirdly, trait anxiety and anxiety disorders can induce somatic symptoms, such as trembling and palpitations (Dayan et al., 2017; Ree et al., 2008). In separate research strands, trait anxiety has been linked to atypical inhibition of motor cortex excitability (Wassermann et al., 2001), decreased activation in sensorimotor regions (Xia et al., 2017), and irregularities in gastric sensorimotor activity (Geeraerts et al., 2005; Van Oudenhove et al., 2007). Altogether, it is plausible that EFE simulation is impacted by the presence of higher levels of trait anxiety due to both atypical emotional and sensorimotor processing.

The present study was designed to address these open questions by exploring both alpha and beta frequency responses to a broad range of EFEs (six basic emotions; Ekman, 1999) in central and noncentral electrode clusters. Unlike previous studies, we presented dynamic rather than static EFEs as the MNS is modulated by the authenticity of biological actions (Gangitano et al., 2004; Ulloa & Pineda, 2007). If different emotions elicit distinct sensorimotor signatures, such desynchronisation patterns should be specific to electrodes overlying sensorimotor cortical regions (versus occipital) and may systematically differ between alpha and beta frequencies. Finally, as a second step, we explored whether potential emotion-specific effects in sensorimotor alpha and beta desynchronisation also systematically differed depending on individual differences in trait anxiety within nonclinical populations.

## Method

### Participants

Thirty healthy adult participants (20 females; mean age = 22.97 years; *SD* = 8.11 years) were recruited from the University of Essex through an online participant recruitment system. A subset of participants (N = 10) was specifically recruited for high trait anxiety through an online prescreen procedure with the State Trait Inventory of Cognitive and Somatic Anxiety (STICSA) questionnaire (Gros et al., 2007; Ree et al., 2008). Participants who were prescreened were included if they scored 43 or higher on STICSA, which indicates a probable case of clinical level anxiety, as suggested by cutoff scores from Van Dam et al. (2013). This was done to ensure a broad range of individual differences in trait anxiety. Participants were compensated with cash or course credits. All participants had normal or corrected-to-normal vision. The study was approved by the University of Essex Ethics Committee, and all participants gave informed, written consent before beginning the experiment.

The sample size was chosen based on Moore et al.’s (2012) study with 30 participants. In addition, a G*Power analysis indicated that at least 24 participants would be required for a medium-to-high effect size (η_p_^2^ = 0.10, α = 0.05, 1-β = 0.80; Erdfelder et al., 2009).

### Materials

Anxiety was measured by using the trait section of the State-Trait Inventory for Cognitive and Somatic Anxiety (STICSA) questionnaire and scores were used for correlation analyses (Gros et al., 2007; Ree et al., 2008). STICSA is a validated self-report measure of anxiety, which is considered more precise than other similar questionnaires, such as the State-Trait Anxiety Inventory and Beck Anxiety Inventory (Carlucci et al., 2018; Gros et al., 2007; Ree et al., 2008). Carlucci et al. (2018) reported that the STICSA questionnaire yielded a hierarchal McDonald omega value (hω) of 0.96 (State) and 0.94 (Trait), which suggests high reliability. Our sample’s STICSA scores showed excellent internal consistency, as demonstrated by Cronbach’s alpha values of 0.90 (cognitive subscale), 0.91 (somatic subscale), and 0.92 (overall trait anxiety, a combination of cognitive and somatic subscales).

The Profile of Mood States (POMS; McNair, 1971), Autistic Spectrum Quotient (ASQ; Baron-Cohen et al., 2001a, b), and Reading the Mind in the Eyes (RTMITE; Baron-Cohen et al., 2001a, b) questionnaires were used to test whether systematic variations in emotion-specific effects in sensorimotor alpha and beta desynchronisation would be specific to anxiety or apply to other emotion-related individual difference constructs.

POMS tested participants’ emotional and mood states on the day of testing (e.g., feeling strongly fatigued, tense, confused, depressed, or angry, as suggested by the POMS subscales; Curran et al., 1995 ; McNair, 1971). We were particularly interested in the tension subscale due to its relation to state anxiety (McNair, 1971).

The ASQ (Baron-Cohen et al., 2001a, b) was used to explore whether trait autism would correlate to sensorimotor alpha or beta desynchronisation, as suggested by past research (Siqi-Liu et al., 2018) and whether this would be modulated by the type of EFE, as ASCs have been associated with emotion recognition difficulties for basic emotions (Fridenson-Hayo et al., 2016).

The RTMITE test was used to explore whether cognitive empathy skills (e.g., theory of mind and social sensitivity skills; Baron-Cohen et al., 2001a, b) would correlate with sensorimotor alpha or beta desynchronisation in response to different EFEs (Curran et al., 1995; McNair, 1971), as a recent meta-analysis showed a moderate correlation between cognitive empathy and MNS activity (Bekkali et al., 2020).

E-Prime 2.0 (Psychology Software Tools, 2015) was used to present emotional stimuli and record keyboard responses. Stimuli were presented full-screen using a Microsoft XP operating system on a 24-inch LCD monitor (1,920 × 1,200 pixels).

The emotional stimuli were a diverse range of dynamic EFE videos acquired online from the MMI Facial Expression Database (Pantic et al., 2005), which were selected to fulfil the following criteria: nonspontaneous emotional expression with action unit information, frontal facing eye contact, same pixel and sample rate, and individuals of different ethnicities and genders to maximise ecological validity.

**Fig. 1:**
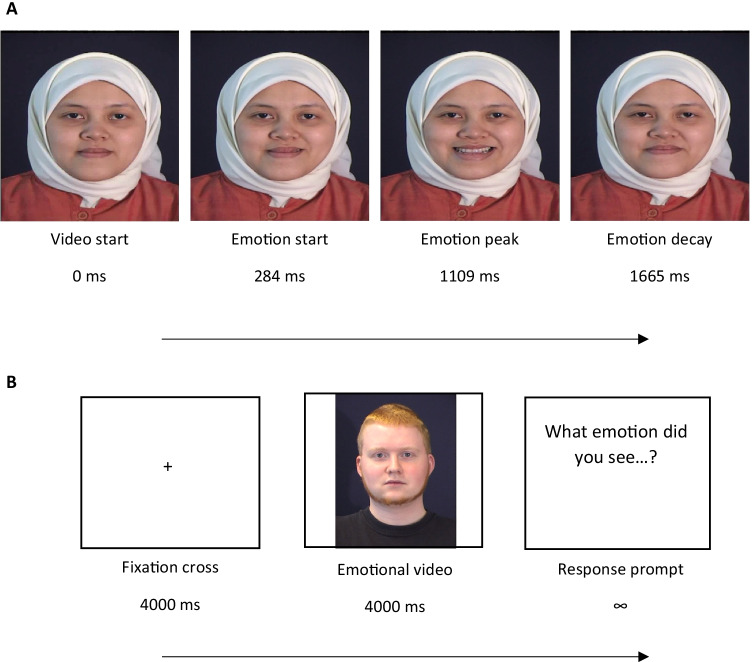
**a** Example of dynamic EFE timeline. Actors began with a neutral face (far left), then preceded to act out the emotion (e.g., zygomaticus major contraction in happy faces), which reached an EFE peak (e.g., smiling) and then the EFE decayed (e.g., zygomaticus major relaxation in happy faces; far right). Times noted reflect average onsets across all conditions. **b** Example of a trial. Each trial started with a fixation cross for a duration of 4*,*000 ms (baseline interval), which was followed by an emotional video for 4*,*000 ms and then participants were prompted for a keyboard press response

Eleven actors (four females) began with a neutral face and then proceeded to act out an emotional expression (Fig. 1a). We used between two and four emotion videos from each actor. Overall, there were five distinct videos of each of the six basic emotions (anger, disgust, fear, happiness, sadness, and surprise). Emotional expressions lasted for an average of 2,490 ms (*SD* = 818 ms), with the average emotional expression beginning at 284 ms (*SD* = 227 ms) and peaking at 1,109 ms (*SD* = 616 ms). See Table 1 in Supplementary Materials for more information on specific emotion conditions.

A post-hoc analysis was conducted to examine whether the emotion videos significantly differed in quantity of motion (as measured by luminance; see Pichon et al., 2009 for details). A one-way ANOVA revealed no significant differences in luminance variation between the emotion levels, *F*(5, 24) = 0.38, *p* = 0.859, η_p_^2^ = 0.073, 1-β = 0.128 (see Table 1 in Supplementary Materials for descriptive statistics).

### Procedure

Participants first completed computerised versions of STICSA, ASQ, RTMITE, and POMS. After this, participants were fitted with an EEG cap and were seated 100-cm away from the screen in a dimly lit room to watch a series of EFEs. After each video, participants provided a report (with no time limit) on which emotion they had seen by pressing a preassigned letter on a computer keyboard (A for an angry face, D for a disgusted face, etc.) before the next trial began (see Fig. 1b for a trial example). Participants watched five blocks of randomly ordered emotion videos. Each block was comprised of 30 trials (five videos per emotion), such that the experiment had a total of 150 trials.

### EEG Recording and Analysis

EEG was recorded using the Neuroscan Synamps2 system and the Scan module of Compumedics Neuroscan (version 4.5.1; 2011) from 64 Ag/AgCl electrodes positioned according to the international 10-10 system (Easycap GmbH, Herrsching, Germany) and referenced online to the left earlobe. Vertical and horizontal electrooculogram (EOG) were recorded from four electrodes placed above and below the midpoint of the right eye (VEOG) and beside the outer canthi of the left and the right eye (HEOG). EEG and EOG were recorded between 0.05 - 100 Hz at a sampling rate of 1,000 Hz.

A low-pass finite impulse response filter of 30 Hz (12 dB roll off) was applied to the data. The filtered data were re-referenced to the common average reference and eye blinks were removed using the Edit module of Compumedics Neuroscan’s (version 4.5.1; 2011) ocular artefact removal method based on Semlitsch et al. (1986). The data were epoched into segments of 8,000 ms, which started with the onset of the fixation cross (4,000-ms duration) and ended 4,000 ms after the onset of the emotion videos. Event-related desynchronisation was calculated based on the following equation: ERD% = (A - R)/R × 100 (where A = frequency power during the presentation of the stimuli and R = reference or baseline before the presentation of the stimuli; see Pfurtscheller & Lopes da Silva, 1999). The fixation cross period was used as the baseline period to provide a sufficient time period for ERD comparison (Pfurtscheller & Lopes da Silva, 1999). The 8,000-ms epochs were trimmed by 500 ms from the start and end of the epoch to reduce warm-up artefacts (Cooper et al., 2013) and then averaged in two frequency bands, alpha (8–12 Hz) and beta (14–28 Hz), with zero phase shift. Amplitudes of single trials were averaged for each emotion condition (i.e., 25 trials contributed to each average) and respectively transformed to percentage changes in alpha and beta power.

**Fig. 2:**
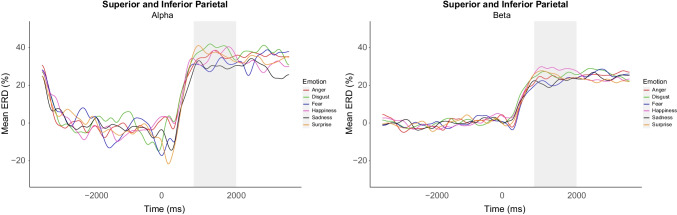
Example of percentage ERD waveforms from the central ROI in both alpha and beta showing visible differences within 800 to 2,000 ms post-stimuli. Positive ERD values show desynchronisation and negative ERD values show event-related synchronisation (ERS)

There was visible desynchronisation in both alpha and beta from around 300 ms after the emotional video onset, corresponding to the mean start of the EFEs within the videos (284 ms). Alpha and beta oscillatory power were extracted for statistical analysis from a window between 800 and 2,000 ms after the emotional video onset. This time window was chosen as it aligned with the average emotional expression peak (1,109 ms) and showed the largest emotion differentiation from visual inspection (Fig. 2).

Scalp electrode sites were divided into two ROIs per hemisphere. The first ROI, superior and inferior parietal (SIP), comprised of central somatosensory MNS regions (Molenberghs et al., 2012). The second ROI (OC), comprised of occipital regions involved in (Perry et al., 2017) visual attention and high-level face perception (Krolak-Salmon et al., 2004). The ROIs were divided in this manner to examine whether EFE-related differences in alpha and beta desynchronisation would be specific to the central regions typically involved in sensorimotor simulation (i.e., SIP) or extend to occipital regions (i.e., OC). This allowed us to delineate whether alpha- and beta-related changes were likely associated with MNS processes, relative to other processes that may be engaged by EFEs (e.g., visual attention; Capotosto et al., 2009; Hobson & Bishop, 2016, 2017; Hopfinger et al., 2000; Vanni et al., 1996). As there is no standard naming system for regions in the brain (Bohland et al., 2009), we identified and described the two ROIs by mapping EEG electrodes to brain regions and their corresponding Brodmann areas, as indicated by Koessler et al. (2009). We also used Molenberghs et al.’s (2012) meta-analysis for guidance in selecting ROIs with mirroring properties.

**Fig. 3:**
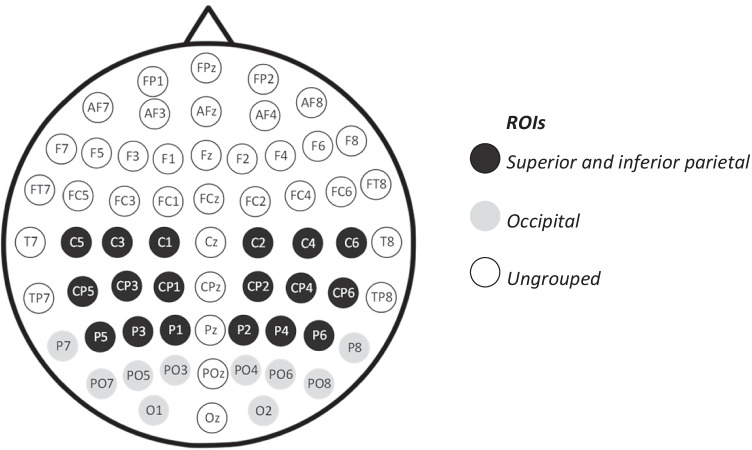
ROIs comprising of central regions (i.e., superior and inferior parietal; SIP) versus other regions (occipital; OC). *Each ROI had a left and right homologue. Please note that ungrouped electrodes were excluded from the analysis*

To reduce the multiple comparison problem (Cooper et al., 2013; Maris & Oostenveld, 2007), the electrodes in each ROI were grouped. Midline (z) electrodes were not included in the analysis. The SIP ROI was formed by grouping superior and inferior parietal electrodes (superior: C1/2, CP1/2, and P1/2 and inferior: C3/4, C5/6, CP3/4, CP5/6, P3/4, and P5/6), and the OC ROI was formed by grouping electrodes surrounding occipitotemporal, posterior parietal, and occipital regions (P7/8, PO3/4, PO5/6, PO7/8, and O1/2), as shown in Fig. 3.

All reported ANOVAs used a 0.05 alpha level; Greenhouse-Geisser or Huynh-Feldt corrected statistics are reported where violations of sphericity assumptions were found.^1^ Reported power calculations were completed post-hoc. All reported pairwise comparisons were Bonferroni-corrected and calculated from estimated marginal means of the relevant factor levels. All reported normality tests were conducted using Shapiro-Wilk tests.

## Results

### Individual Differences in Trait Anxiety, Trait Autism, Cognitive Empathy, and Mood States

STICSA scores (*M* = 41.67, *SD* = 12.45; range 25 - 73) were normally distributed (*p* > 0.05). Twelve of our 30 participants (eight females) had a STICSA score of 43 or above, which is the cutoff score for high trait anxiety (Van Dam et al., 2013). The remaining 18 participants (12 females) scored below this cutoff. This shows that we successfully recruited across a broad range of trait anxiety scores for the purpose of systematically investigating anxiety’s modulating effects on sensorimotor alpha and beta desynchronisation responses to EFEs.

ASQ scores (*M* = 20.13, *SD* = 5.72; range 4 - 30) were normally distributed (*p* > 0.05). All participants were classed as low trait autism participants, because they did not score above the suggested cutoff score of 36 (Baron-Cohen et al., 2001a, b). RTMITE scores (*M* = 27.50, *SD* = 3.43; range 20 - 35) were normally distributed (*p* > 0.05). Normality for POMS scores differed depending on the subscale. Scores were non-normal for the total disturbance score (*M* = 25.00, *SD* = 29.55; range -14 to 113; *p* = 0.014) and the following POMS subscales: tension (*M* = 8.50, *SD* = 6.67; range 1 - 25; *p* = 0.001), depression (*M* = 7.47, *SD* = 9.85; range 0 - 41; *p* < 0.001), and anger (*M* = 4.67, *SD* = 6.74; range 0 - 26; *p* < 0.001). However, POMS subscales for fatigue (*M* = 6.73, *SD* = 4.97; range 0 - 18; *p* = 0.078), confusion (*M* = 7.60, *SD* = 4.48; range 0 - 18; *p* = 0.48), and vigour (*M* = 9.97, *SD* = 5.27; ranging from 1 to 22; *p* = 0.613) were normally distributed. Nonparametric tests were conducted for scales with non-normal distributions.

### Behavioural Performance

Participants’ overall accuracy at identifying each emotion was reasonably high (*M* = 83.44%, *SD* = 7.59) with happy faces being recognised at the highest rate (*M* = 97.75%, *SD* = 5.07) and fearful faces being recognised at the lowest rate (*M* = 65.93%, *SD* = 22.26).^2^ A one-way ANOVA was conducted to examine whether emotion recognition accuracy was influenced by emotion type. A significant main effect of emotion was found (*F*(5, 140) = 24.20, *p* < 0.001, η_p_^2^ = 0.464, 1-β = 1.00). Of the 15 emotion pairings, only 4 did not significantly differ (anger vs. sadness, disgust vs. happiness, disgust vs. surprise, and happiness vs. surprise; |*t*(14)| ≤ 2.15, *p* ≥ 0.504), which suggests that emotion recognition accuracy varied depending on the dynamic emotion displayed (Tables 2 and 3 in Supplementary Materials for full descriptive statistics and pairwise comparisons, respectively).

Pearson’s and Spearman’s correlation tests did not show consistent significant relationships between emotion recognition accuracy scores and individual difference measures (Table 4 in the Supplementary Materials for full results). This suggests that individual differences in trait anxiety, mood states, autism, and cognitive empathy did not significantly impact the overt ability to identify facial emotions in the emotional videos.

### Emotion-related Differences in Beta, but not Alpha, Desynchronisation are Specific to Central ROI

For alpha and beta oscillatory activity separately, we conducted a 2 (ROI: SIP and OC) × 6 (emotion: anger, disgust, fear, happiness, sadness and surprise) × 2 (hemisphere: left and right) three-way repeated-measures ANOVA to investigate whether emotion-related differences were specific to central regions.

For alpha, there were no significant emotion-related main or interaction effects of interest, *F*(5, 145) ≤ 1.63, *p* ≥ 0.189, η_p_^2^ = ≤ 0.053, 1-β ≤ 0.413. For beta, there was a significant main effect of emotion, *F*(5, 145) = 2.86, *p* = 0.017, η_p_^2^ = 0.090, 1-β = 0.831, and a significant interaction effect between ROI and emotion, *F*(5, 145) = 4.26, *p* = 0.001, η_p_^2^ = 0.128, 1-β = 0.957.

Therefore, one-way repeated-measures ANOVAs tested effects of emotion separately for each ROI. The left and right hemispheres were collapsed as there were no significant interaction effects involving hemisphere in the above analysis.

**Fig. 4:**
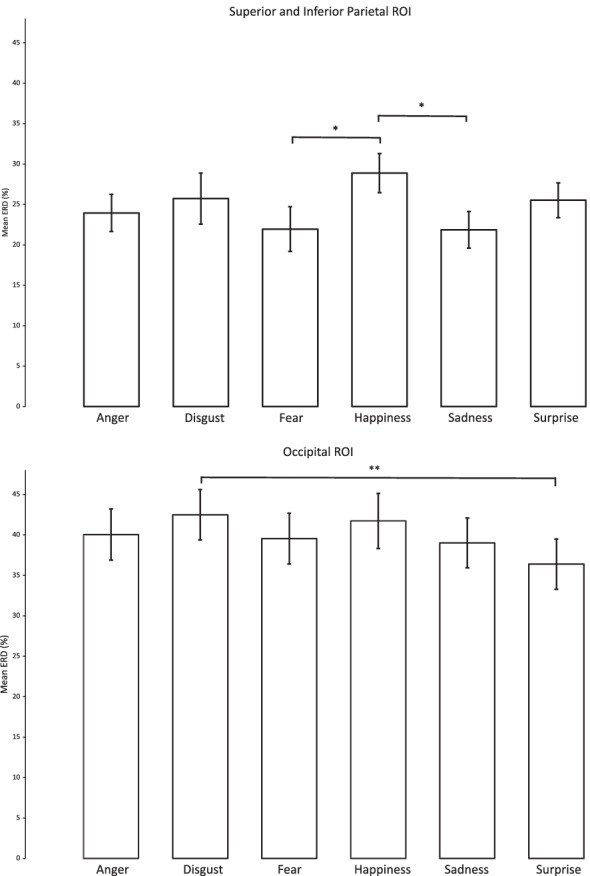
Beta mean percentage change in both ROIs in response to the six basic emotions. Each bar represents collapsed activity from the left and right hemisphere. Significant pairwise comparisons: *p < 0.05 and **p ≤ 0.01. Error bars represent the standard error of the mean ERD

For the central (SIP) ROI, a significant main effect of emotion was found, *F*(5, 145) = 3.15, *p* = 0.010, η_p_^2^ = 0.098, 1-β = 0.871. Pairwise comparisons showed that happiness elicited significantly higher beta desynchronisation than fear (*p* = 0.020) and sadness (*p* = 0.017; Fig. 4). None of the other emotion pairings were significantly different (*p* ≥ 0.108).

For the occipital (OC) ROI, a significant main effect of emotion was also found, *F*(5, 145) = 3.25, *p* = 0.008, η_p_^2^ = 0.101, 1-β = 0.882. Pairwise comparisons showed that disgust elicited significantly higher beta desynchronisation than surprise (*p* = 0.009; Fig. 4). None of the other emotion pairings were significantly different (*p* ≥ 0.169).

The spatial pattern of emotion-related differences in beta ERDs in central and occipital ROIs support the hypothesis that differentiation between happiness, sadness, and fear reflects MNS activity rather than visual attention during emotion observation, whereas differentiation between disgust and surprise may indicate their relative visual attentional engagement.

**Fig. 5:**
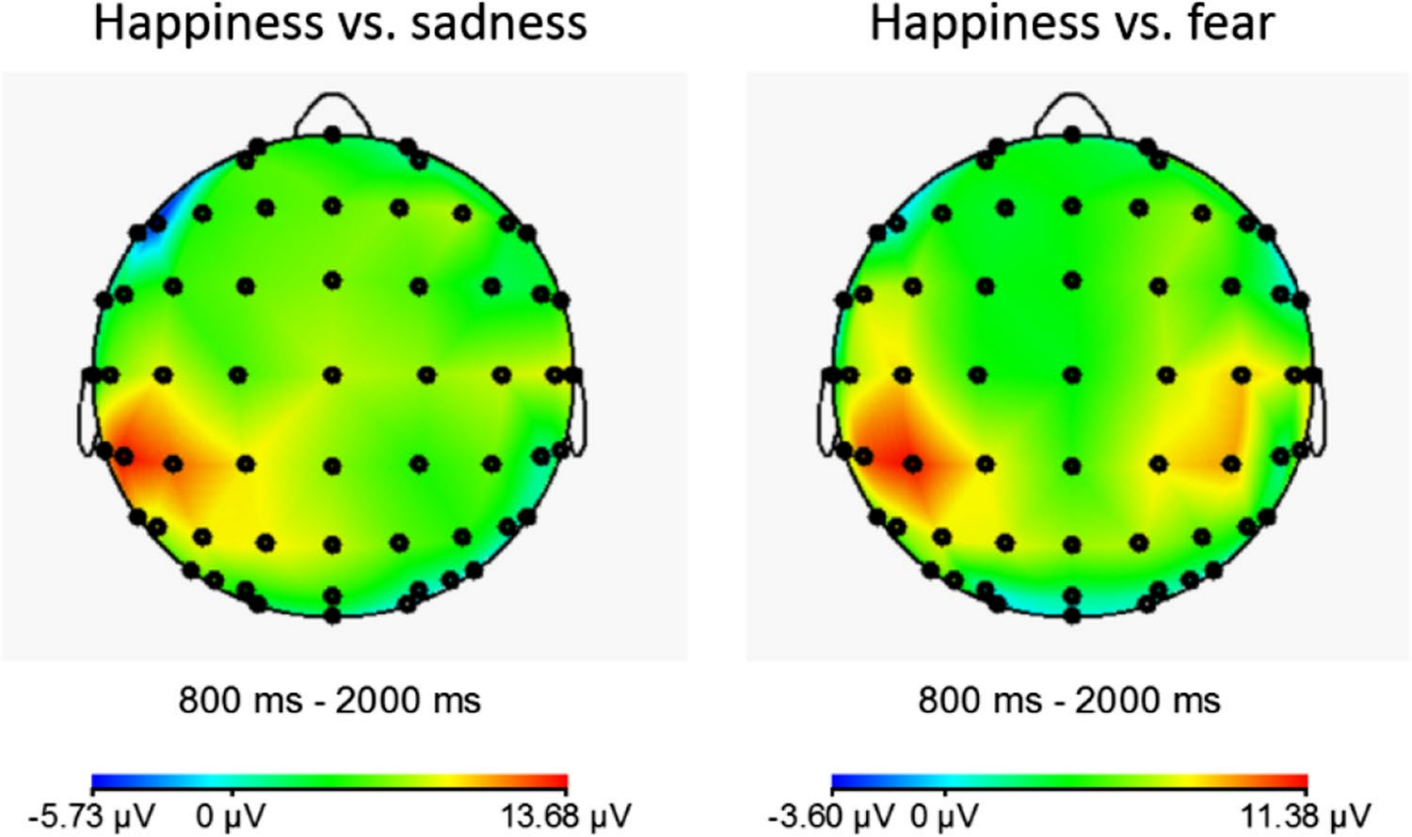
Scalp topographies of beta ERD difference waveforms (left panel: happiness minus sadness; right panel: happiness minus fear) in the 800-2,000 ms time window after EFE onset

To further explore this assumption, we calculated difference waveforms of the significant emotional pairings found in the central ROI analysis by subtracting one emotion’s beta ERD waveforms from the other emotion’s beta ERD waveforms (happiness minus sadness; happiness minus fear). Scalp topographies of these difference waveforms illustrate that the largest differences in the 800 - 2,000 ms time window occurred at centroparietal electrodes, that is, within our central ROIs (Fig. 5). Source localisations on these difference waveforms were completed using LORETA in BrainVision Analyzer 2 (Version 2.2.0, Brain Products GmbH, Gilching, Germany). ROIs were voxel clusters within 21 predefined Brodmann areas (BA) plus amygdala and hippocampus regions separately for left and right hemispheres (for more information on the voxels used see https://www.brainproducts.com/files/public/downloads/LORETA/All_ROIs.xml). The largest current source densities within the 800 - 2,000 ms time window were seen for the 5 voxels defining BA2 in the left hemisphere for the happiness versus sadness difference wave (peak density: 0.22 V at 1,209 ms) and for the 4 voxels defining BA1 in the right hemisphere for the happiness versus fear difference wave (peak density: 0.16 V at 1,497 ms). This suggests that the strongest neural sources of emotional differentiation between happiness, sadness, and fear may be found in the portions of primary somatosensory cortex that have mirroring properties (BA1 and 2; Keysers et al., 2010) and that are thought to be the strongest source of the mu rhythm (BA2; Salmelin & Hari, 1994; see also Hamilton, 2013).

### Central Beta Desynchronisation Relates to Individual Differences in Trait Anxiety and Trait Autism

Pearson’s and Spearman’s correlation tests explored the relationship between emotion-related beta desynchronisation for the central (SIP) ROI (collapsed across hemispheres) and individual differences in trait anxiety (STICSA), as per our aims, but also in POMS subscales, trait autism (ASQ) and cognitive empathy (RTMITE).

Difference beta ERD waveforms (happiness minus sadness; happiness minus fear) were entered into correlational analyses with the scores from each individual difference measure.

**Fig. 6:**
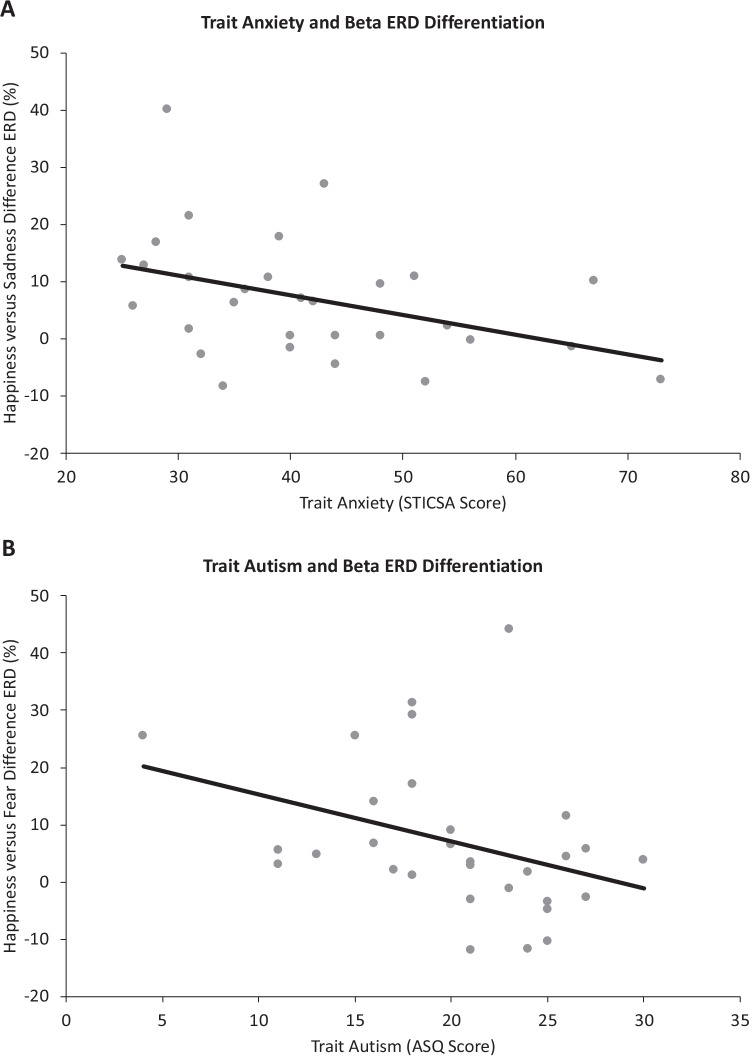
**a** Scatterplot shows a medium negative correlation between trait anxiety and happiness versus sadness difference ERD for the SIP ROI within the beta frequency band. Positive values on the y-axis represent where ERD in the happiness condition was greater than in the sadness condition. Negative values on the y-axis represent where ERD in the sadness condition was greater than in the happiness condition. Each point represents a participant. **b** Scatterplot shows a medium negative correlation between trait autism and happiness versus fear difference ERD for the SIP ROI within the beta frequency band. Positive values on the y-axis represent where ERD in the happiness condition was greater than in the fear condition. Negative values on the y-axis represent where ERD in the fear condition was greater than in the happiness condition. Each point represents a participant

Correlation tests showed a medium-strength negative relationship between the trait anxiety scores and happiness versus sadness difference ERD (Pearson’s r = -0.40, *p* = 0.027; Fig. 6a). In other words, individuals with higher trait anxiety showed smaller differences in central beta desynchronisation to viewing happy versus sad EFEs, suggesting that such individuals simulate these two emotions more similarly (i.e., differentiate these emotions to a lesser extent) in their sensorimotor system than less anxious individuals.

There also was a medium-strength negative relationship between ASQ scores and happiness versus fear ERD differentiation (Spearman’s r = -0.42, *p* = 0.020). In other words, individuals with higher trait autism showed smaller differences in central beta desynchronisation to viewing happy versus fearful EFEs in the SIP ROI, suggesting that such individuals simulate these two emotions more similarly (i.e., differentiate these emotions to a lesser extent) in their sensorimotor system than lower-trait autistic individuals (Fig. 6b). No other correlations were significant (see Table 4 in Supplementary Materials for full correlation results). It is noteworthy that difference ERDs were related to trait anxiety (STICSA) but not state anxiety (POMS tension), which suggests that weakened sensorimotor differentiation may be a chronic rather than a transient feature of high anxiety.

## Discussion

The purpose of the present study was to delineate systematic differences in MNS sensorimotor activations, as measured by alpha and beta desynchronisation over central (versus occipital) scalp regions, in response to a broad range of emotional facial expressions. Our study is the first to attempt to describe how the sensorimotor MNS delineates the six basic emotions with dynamic stimuli. Additionally, our study expanded for the first time on how such emotional differentiation may relate to individual differences in trait anxiety in a nonclinical adult population.

### Sensorimotor Simulation of Happy Versus Fearful and Sad Faces

Significant differences between happiness and fear and between happiness and sadness were seen bilaterally at the superior and inferior parietal ROI. These differences were source localised to somatosensory cortical regions thought to be involved in the simulation of emotions (Adolphs et al., 2000; Moore et al., 2012). Specifically, dynamic facial expressions of happiness resulted in greater beta desynchronisation compared to expressions of sadness and fear. These effects did not extend to occipital scalp regions and were specific to oscillatory activity in the beta band; alpha desynchronisation did not reliably distinguish between different emotional expressions at any ROI. Altogether, this suggests that our emotional effects for happiness, sadness, and fear reflect genuine differentiations in the sensorimotor component of action observation rather than the differential engagement of visual attention, which is typically marked by more posterior alpha desynchronisation (Angelini et al., 2018; Hobson & Bishop, 2017; Klimesch, 2012; Salmelin & Hari, 1994).

We also observed an emotion differentiation specific to the occipital ROI: dynamic facial expressions of disgust resulted in greater beta desynchronisation compared to expressions of surprise. We propose that stronger ERD responses to disgusted EFEs may indicate greater allocation of attentional resources as an important survival mechanism related to avoidance (Curtis et al., 2004; Oaten et al., 2009). Vanni et al. (1996) argued that the occipital processing of attentionally engaging stimuli interacts with frontoparietal attention networks to drive orienting responses and further visual processing. Disgust stimuli may be particularly engaging of this attentional mechanism, as suggested by our findings. Importantly, however, such posterior differentiation did not occur for happiness versus sadness or fear, showing that selective visual attention cannot explain central emotion-specific effects in beta desynchronisation for happy, sad, and fearful facial expressions.

Overall, our findings support the suggestion that the extended MNS is able to distinguish between various EFEs to some degree (Capotosto et al., 2009; Hopfinger et al., 2000; Moore et al., 2012; Vanni et al., 1996) and show that this distinction is measurable through mu oscillatory activity.^3^^,^^4^ The clearest sensorimotor distinction we observed appeared to support valence and action motivation models of emotion relative to the discrete model, as the observed significant differences were between an emotion associated with positive and approach tendencies (happiness) and emotions associated with negative and withdrawal tendencies (fear and sadness; Buss et al., 2003; Coan et al., 2001; Harmon-Jones & Allen, 1998; Lane et al., 1997). This is consistent with Moore et al.’s (2012) findings, which previously showed differences between happy (i.e., positive/approach) and disgusted (i.e., negative/withdrawal) faces within central electrodes in sensorimotor alpha. It is important to note that Moore et al. (2012) used static EFEs whereas the current study used dynamic EFEs that may have contributed to some of the discrepancies in the emotion effects. Dynamic EFEs may provide greater temporal authenticity (Gangitano et al., 2004), increase activation in premotor cortex (Sato et al., 2004), and elicit oscillatory changes, particularly in the beta range (Pavlidou et al., 2014; Pfurtscheller & Lopes da Silva, 1999). Our study shows that beta may be the sensorimotor currency for distinguishing valence or motivational value of dynamic EFEs, whereas alpha may contribute more to distinguishing between static EFEs.

Greater desynchronisation for happy faces compared with negative faces is consistent with behavioural studies showing that higher levels of mimicry are linked to prosocial behaviours (van Baaren et al., 2004). For example, increased motivation to mimic happiness over sadness may derive from the notion that happy faces are more likely to signal altruistic and prosocial behaviours relative to sad faces and may thus be attributed with higher rewards (e.g., prosocial interactions) than sad faces (Hess & Fischer, 2014; Schmidt & Cohn, 2001; Trivers, 1971).

Sensorimotor simulation of happiness also could be favoured due to the “happy face advantage” whereby happy faces are detected more accurately than negative faces, such as sad or fearful faces (Kirita & Endo, 1995; Svärd et al., 2012; Wells et al., 2016). Although there may be an evolutionary advantage in detecting negative emotions (Vaish et al., 2008), it is suggested that happy faces may be detected more accurately due to their unique facial feature of smiling (U-shaped lips; Kirita & Endo, 1995). This unique facial feature of smiling seen in happy faces may be easier to simulate and distinguish in sensorimotor regions, leading to heightened mu desynchronisation compared with negative expressions, such as fear and sadness, which often require additional facial information to decode the expression (Adolphs, 2002; Calvo & Beltrán, 2014). Furthermore, the happy face advantage could explain why happy faces had the highest emotion recognition accuracy in our study.

Additionally, increased desynchronisation for happy relative to fearful and sad faces may reflect emotion regulation processes. Within the framework of the facial feedback hypothesis (Soussignan, 2002), motor executions associated with positive facial expressions may enhance a positive affective state (Sel et al., 2015); therefore, the heightened mu desynchronisation for happy faces could be motivated by a need to augment positive feelings through increasing the simulation of positive motor actions.

Whilst these findings support theories of emotion recognition through sensorimotor simulation, there was less clear evidence for MNS emotional specificity being completely based on discrete attributes, as not all basic emotions had distinct mu ERD profiles. Moreover, our findings do not provide outright support for the right lateralisation of emotion-related responses in the sensorimotor region (Adolphs et al., 2000; Rayson et al., 2016), as no modulatory effects of hemisphere were observed.

### Trait Anxiety and Autism Attenuate Differential Simulation of Emotion

Our study further demonstrated that individual differences in trait anxiety and autism may systematically modulate or be modulated by mu oscillatory responses to EFEs. These findings are in line with an individual differences account of emotion mirroring and provide further evidence to the broader notion that atypical sensorimotor activity is associated with difficulties in emotional and social processing (Cooper et al., 2013; Siqi-Liu et al., 2018). Our findings therefore have important implications for future studies of subclinical and clinical populations with anxiety and ASCs.

We observed a negative relationship between trait anxiety scores and the differentiation of happiness and sadness within central beta desynchronisation. These findings suggest that the sensorimotor simulation of happy and sad facial expressions is less distinct the higher a person’s anxiety levels are, despite the uniqueness of happy facial expressions and their dimensional opposition to sadness. This is consistent with research showing that, compared with controls, individuals with generalised anxiety disorder show differences in resting-state activity within sensorimotor and emotion processing regions (Xia et al., 2017). Whilst STICSA scores do not control for combined anxiety and depression, our findings echo those of Berg et al. (2016), who suggested that those with combined anxiety and depression have less sensitivity to happy and sad faces, because they needed more emotional intensity to accurately recognise an EFE relative to those with nonanxious depression.

More broadly, these findings support suggestions that lower emotion discrimination skills (i.e., not being able to discern between different emotions) are closely linked to poor emotional wellbeing, increased stress levels (Erbas et al., 2018; Lennarz et al., 2018), social anxiety disorder, and depression (Demiralp et al., 2012; Kashdan & Farmer, 2014). Our findings therefore extend MNS literature from depression, schizophrenia, and ASCs to include individual differences in anxiety. Furthermore, showing that anxiety levels modulated sensorimotor beta desynchronisation in response to dynamic EFEs provides early evidence that could have important implications for the prevention and treatment of anxiety disorders. Within the general population, those with higher trait anxiety and higher neuroticism have a greater risk of developing clinical anxiety disorders, which has been attributed to the increasing cognitive tendencies (e.g., worrying and negative cognitive biases) that are heightened in anxiety disorders (Barlow et al., 2014; Gawda & Szepietowska, 2016; Lengel et al., 2016; Wang et al., 2019). Sensorimotor simulation could be used to explore and measure the effects of alternative anxiety treatments, such as NFT targeting MNS regions, at stages that precede overt behavioural difficulties.

Systemic modulation of sensorimotor simulation were not limited to trait anxiety in our study. We also found a medium-strength negative relationship between trait autism and the differentiation of happiness and fear in central beta ERD, which suggests that the differentiation between happy and fearful facial expressions may be weakened in those with higher trait autism. This finding is consistent with past research linking the emotional and social difficulties related to ASCs to atypical mu desynchronisation (Siqi-Liu et al., 2018). The reduced distinction between happy and fearful faces may be related to suggestions that ASCs may result in emotion recognition difficulties specific to happy (Sato et al., 2017) and fearful faces (Howard et al., 2000; Humphreys et al., 2007; Pelphrey et al., 2002), and extends them to a population with a subclinical range of autistic spectrum traits. However, other ASC studies have argued against a specific emotion recognition deficit for happiness or fear (Griffiths et al., 2019; Rump et al., 2009; Shanok et al., 2019; Sucksmith et al., 2013). Given these inconsistent findings, more research is needed to clarify whether subdued sensorimotor activity contributes to the emotion recognition difficulties experienced by some individuals with ASCs.

### Limitations and Future Directions

Our study provided a first account of trait anxiety and mu desynchronisation from a discrete emotion perspective (basic emotions; Ekman & Friesen, 1971). Future research could focus on contrasting dimensional (e.g., valence and arousal; Russell, 1980; Watson & Tellegen, 1985) and action motivation accounts of emotional simulation (Coan et al., 2001; Corr, 2013). It also is possible that mu suppression patterns for different emotions may be better accommodated by other theoretical perspectives, such as the negative potentiation, positive attenuation, and emotion context insensitivity (ECI) hypotheses (Bylsma et al., 2008; Rottenberg et al., 2005). From these perspectives, it is possible that mu desynchronisation in more anxious participants may be either heightened (indexing stronger simulation) for negatively valenced EFEs (negative potentiation hypothesis) or weakened (indexing weaker simulation) for positively valenced EFEs (positive attenuation hypothesis; Bylsma et al., 2008; Rottenberg et al., 2005) relative to less anxious participants. Alternatively, more anxious individuals may simulate emotions to a lesser extent than less anxious individuals regardless of the valence or the discrete significance of the emotion, leading to weaker mu desynchronisation for all EFEs (ECI hypothesis; Attwood et al., 2017; Bylsma et al., 2008; Rottenberg et al., 2005; Rottenberg & Hindash, 2015). Although mainly discussed within the context of depression, these hypotheses may apply to anxiety by virtue of the significant comorbidity and symptom overlap between these emotional disorders (Goodwin, 2015; Hirschfeld, 2001).

Beyond theoretical limitations, using a baseline that shows a neutral face- and/or movement (rather than a fixation cross) could help to ensure that desynchronisation is due to genuine EFE simulations rather than the desynchronising effects of general (facial) movements. However, neutral dynamic face baselines may not be optimal as it has been suggested that neutral or emotionally ambiguous faces can increase sensorimotor activity (Karakale et al., 2018; Krivan et al., 2020) and may therefore elicit emotionally relevant modulation of mu frequencies. Baseline intervals containing scrambled versions of EFEs may be a better choice as this reduces the emotional salience of stimuli while retaining lower-level visual features including movement (Orgs et al., 2008; Rayson et al., 2016). Alternatively, studies could compare the effects of different baselines (e.g., static or dynamic biological movement; nonbiological/scrambled movement) to delineate their effects on mu frequencies. Certain mu frequencies (e.g., low beta) may be more affected by the type of baseline relative to other mu frequencies (e.g., alpha; Puzzo et al., 2011). Future research could extend Puzzo et al.'s (2011) findings to investigate this issue within the context of emotional facial stimuli.

## Conclusion

The current study provided a detailed account of how the MNS may be involved in the differential recognition of the six basic emotions and demonstrated the involvement of sensorimotor simulation in the distinction of emotions, such as happiness, sadness, and fear. Our findings also suggest that the presence of trait anxiety and trait autism may systematically affect how well sensorimotor regions distinguish between happy and sad, and happy and fearful dynamic facial expressions, respectively. Taken together, this study emphasises the probable involvement of the MNS in emotion differentiation (Moore & Franz, 2017). To our current knowledge, this is the first study to explore and describe how sensorimotor alpha and beta oscillatory activity (i.e., mu) differentiates between the six basic emotions with the use of dynamic emotional stimuli. Additionally, our findings provide electrophysiological evidence for the possibility that emotional difficulties related to anxiety may be compounded by weakened sensorimotor simulation.

## Supplementary Information

ESM 1 (DOCX 45 kb)

## Footnotes

1 Analyses conducted with BrainVision Analyzer 2 (2019), IBM SPSS (2021) and JASP (2021)

2 One participant’s emotion recognition accuracy scores were excluded due to data recording issues; therefore, the accuracy analyses were based on a sample of 29 healthy adult participants (20 females; mean age = 22.93 years; SD = 8.26 years).

3 It is possible that the temporal variability of the videos could have some impact on our findings; however, it is important to mention that all emotion conditions, except for surprise, had emotional peaks within the selected time window, which suggests that the ERD calculations included the most intense movements of each emotion for almost every condition. Furthermore, the patterns of temporal variability (e.g., happiness peaking earlier than sadness and fear, the three emotions which differed in central beta ERD) are unlikely to account for our findings, as other emotions (e.g., surprise and anger) also peaked early but did not cause comparable ERD effects.

4 Whilst low-level visual aspects, such as quantity of motion and luminance, may modulate brain activity within various frequencies (including alpha and beta), locations (including central and parietal regions) and at specific latencies in response to emotional stimuli (Allaert et al., 2020; Eroğlu et al., 2020, 2022; Quadrelli et al., 2019), it is unlikely that differences in these low-level visual aspects would have contributed to the significant effects seen between happy and sad faces, as the emotion videos did not significantly differ in quantity of motion as assessed by luminance variation (see Pichon et al., 2009, for details).
