# Supplementary material for: Differential beta desynchronisation responses to dynamic emotional facial expressions are attenuated in higher trait anxiety and autism
Source: Cogn Affect Behav Neurosci. 2022 Jun 27;22(6):1404–20. doi: 10.3758/s13415-022-01015-x (PMC9622532; doi:10.3758/s13415-022-01015-x)
Supplement: Supplementary file 1 — (DOCX 45 kb) [file 13415_2022_1015_MOESM1_ESM.docx]

**Supplementary Materials**

***Emotional facial expression video attributes***

| **Table 1**  *Emotional facial expression video attributes (ms)* | | | | | | | |
| --- | --- | --- | --- | --- | --- | --- | --- |
|  |  | Luminance variation | Emotion start | Emotion peak | Emotion decay | Emotion end | Emotion duration |
| Overall | *M* | 21.68 | 284 | 1109 | 1665 | 2774 | 2490 |
|  | *SD* | 2.35 | 226.60 | 615.83 | 840.80 | 823.39 | 818.37 |
| Anger | *M* | 21.76 | 272 | 840 | 2577 | 3417 | 3145 |
|  | *SD* | 3.79 | 163 | 362 | 962 | 803 | 701 |
| Disgust | *M* | 21.53 | 288 | 1192 | 1592 | 2784 | 2496 |
|  | *SD* | 2.16 | 39 | 559 | 470 | 566 | 576 |
| Fear | *M* | 20.53 | 432 | 1144 | 1813 | 2957 | 2525 |
|  | *SD* | 2.02 | 406 | 553 | 779 | 472 | 513 |
| Happiness | *M* | 21.54 | 320 | 1008 | 1277 | 2285 | 1965 |
|  | *SD* | 1.06 | 225 | 458 | 488 | 847 | 818 |
| Sadness | *M* | 22.29 | 128 | 1752 | 1450 | 3202 | 3074 |
|  | *SD* | 3.06 | 64 | 762 | 607 | 340 | 339 |
| Surprise | *M* | 22.45 | 264 | 720 | 1280 | 2000 | 1736 |
|  | *SD* | 1.89 | 48 | 72 | 733 | 665 | 632 |

***Behavioural data***

| **Table 2**  *Emotion accuracy scores (%)* | | |
| --- | --- | --- |
|  | *M* | *SD* |
| Overall | 83.44 | 7.59 |
| Anger | 76.83 | 15.36 |
| Disgust | 92.42 | 13.58 |
| Fear | 65.93 | 22.26 |
| Happiness | 97.75 | 5.07 |
| Sadness | 77.39 | 13.85 |
| Surprise | 90.35 | 8.72 |

| **Table 3**  *Emotion accuracy scores pairwise comparisons (Bonferroni-corrected)* | | | | | |
| --- | --- | --- | --- | --- | --- |
|  |  | Mean Difference | *SE* | *t* | *p* |
| Anger | Disgust | -15.59*** | 3.45 | -4.52 | < .001 |
|  | Fear | 10.90* | 3.45 | 3.16 | 0.029 |
|  | Happiness | -20.92*** | 3.45 | -6.06 | < .001 |
|  | Sadness | -0.57 | 3.45 | -0.16 | 1.000 |
|  | Surprise | -13.52** | 3.45 | -3.92 | 0.002 |
| Disgust | Fear | 26.49*** | 3.45 | 7.68 | < .001 |
|  | Happiness | -5.33 | 3.45 | -1.54 | 1.000 |
|  | Sadness | 15.03*** | 3.45 | 4.36 | < .001 |
|  | Surprise | 2.07 | 3.45 | 0.60 | 1.000 |
| Fear | Happiness | -31.82*** | 3.45 | -9.22 | < .001 |
|  | Sadness | -11.46* | 3.45 | -3.32 | 0.017 |
|  | Surprise | -24.41*** | 3.45 | -7.08 | < .001 |
| Happiness | Sadness | 20.35*** | 3.45 | 5.90 | < .001 |
|  | Surprise | 7.40 | 3.45 | 2.15 | 0.504 |
| Sadness | Surprise | -12.95** | 3.45 | -3.75 | 0.004 |

* *p* < .05, ** *p* < .01 and *** *p* < .001.

***Correlations between individual difference measures, emotion accuracy scores and beta difference ERD***

**Table 4**

*Correlations Between Individual Difference Scores, Emotion Recognition Scores (%) and Beta Difference ERD*

| Variable |  | 1 | 2 | 3 | 4 | 5 | 6 | 7 | 8 | 9 | 10 | 11 | 12 | 13 | 14 | 15 | 16 | 17 | 18 | 19 |
| --- | --- | --- | --- | --- | --- | --- | --- | --- | --- | --- | --- | --- | --- | --- | --- | --- | --- | --- | --- | --- |
| 1. STICSA | r | — |  |  |  |  |  |  |  |  |  |  |  |  |  |  |  |  |  |  |
|  | *p* | — |  |  |  |  |  |  |  |  |  |  |  |  |  |  |  |  |  |  |
| 2. ASQ | r | 0.29 | — |  |  |  |  |  |  |  |  |  |  |  |  |  |  |  |  |  |
|  | *p* | 0.116 | — |  |  |  |  |  |  |  |  |  |  |  |  |  |  |  |  |  |
| 3. RTMITE | r | -0.24 | -0.18 | — |  |  |  |  |  |  |  |  |  |  |  |  |  |  |  |  |
|  | *p* | 0.197 | 0.348 | — |  |  |  |  |  |  |  |  |  |  |  |  |  |  |  |  |
| 4. POMS (Total Disturbance) | r | 0.84******* | 0.20 | -0.04 | — |  |  |  |  |  |  |  |  |  |  |  |  |  |  |  |
|  | *p* | < .001 | 0.279 | 0.826 | — |  |  |  |  |  |  |  |  |  |  |  |  |  |  |  |
| 5. POMS (Tension) | r | 0.81******* | 0.23 | -0.12 | 0.92******* | — |  |  |  |  |  |  |  |  |  |  |  |  |  |  |
|  | *p* | < .001 | 0.225 | 0.522 | < .001 | — |  |  |  |  |  |  |  |  |  |  |  |  |  |  |
| 6. POMS (Depression) | r | 0.66******* | 0.10 | -0.08 | 0.87******* | 0.88******* | — |  |  |  |  |  |  |  |  |  |  |  |  |  |
|  | *p* | < .001 | 0.591 | 0.687 | < .001 | < .001 | — |  |  |  |  |  |  |  |  |  |  |  |  |  |
| 7. POMS (Anger) | r | 0.26 | -0.31 | -0.21 | 0.59******* | 0.53** | 0.64******* | — |  |  |  |  |  |  |  |  |  |  |  |  |
|  | *p* | 0.163 | 0.092 | 0.27 | < .001 | 0.003 | < .001 | — |  |  |  |  |  |  |  |  |  |  |  |  |
| 8. POMS (Fatigue) | r | 0.59******* | 0.17 | -0.14 | 0.79******* | 0.61******* | 0.65******* | 0.58******* | — |  |  |  |  |  |  |  |  |  |  |  |
|  | *p* | < .001 | 0.369 | 0.449 | < .001 | < .001 | < .001 | < .001 | — |  |  |  |  |  |  |  |  |  |  |  |
| 9. POMS (Confusion) | r | 0.8******* | 0.30 | -0.25 | 0.86******* | 0.80******* | 0.61******* | 0.35 | 0.63******* | — |  |  |  |  |  |  |  |  |  |  |
|  | *p* | < .001 | 0.102 | 0.184 | < .001 | < .001 | < .001 | 0.061 | < .001 | — |  |  |  |  |  |  |  |  |  |  |
| 10. POMS (Vigour) | r | -0.42***** | -0.30 | -0.25 | -0.44***** | -0.3 | -0.16 | 0.25 | -0.23 | -0.39***** | — |  |  |  |  |  |  |  |  |  |
|  | *p* | 0.02 | 0.106 | 0.178 | 0.016 | 0.111 | 0.398 | 0.186 | 0.228 | 0.032 | — |  |  |  |  |  |  |  |  |  |
| 11. Overall Accuracy | r | -0.02 | -0.26 | 0.27 | 0.05 | 0.06 | 0.08 | 0.11 | 0.02 | 0.19 | -0.07 | — |  |  |  |  |  |  |  |  |
|  | *p* | 0.928 | 0.174 | 0.156 | 0.792 | 0.746 | 0.696 | 0.585 | 0.928 | 0.314 | 0.715 | — |  |  |  |  |  |  |  |  |
| 12. Anger Accuracy | r | 0 | -0.29 | 0.10 | -0.03 | 0.05 | 0.03 | 0.12 | 0.03 | 0.05 | 0.20 | 0.72******* | — |  |  |  |  |  |  |  |
|  | *p* | 0.995 | 0.122 | 0.6 | 0.861 | 0.815 | 0.871 | 0.525 | 0.891 | 0.807 | 0.29 | < .001 | — |  |  |  |  |  |  |  |
| 13. Disgust Accuracy | r | 0.01 | -0.38***** | 0.21 | 0.18 | 0.01 | 0.04 | 0.19 | 0.15 | 0.11 | -0.23 | 0.46***** | 0.24 | — |  |  |  |  |  |  |
|  | *p* | 0.955 | 0.044 | 0.272 | 0.351 | 0.955 | 0.858 | 0.313 | 0.429 | 0.567 | 0.234 | 0.012 | 0.221 | — |  |  |  |  |  |  |
| 14. Fear Accuracy | r | -0.04 | -0.33 | 0.44***** | 0.13 | 0.15 | 0.16 | 0.26 | 0.12 | 0.07 | 0.08 | 0.75******* | 0.35 | 0.37***** | — |  |  |  |  |  |
|  | *p* | 0.846 | 0.082 | 0.018 | 0.52 | 0.434 | 0.396 | 0.174 | 0.524 | 0.706 | 0.697 | < .001 | 0.067 | 0.047 | — |  |  |  |  |  |
| 15. Happy Accuracy | r | 0.43***** | 0.22 | 0.07 | 0.40***** | 0.44***** | 0.46***** | 0.06 | 0.25 | 0.45***** | -0.15 | 0.28 | 0.11 | -0.05 | 0.29 | — |  |  |  |  |
|  | *p* | 0.02 | 0.252 | 0.732 | 0.033 | 0.016 | 0.012 | 0.755 | 0.183 | 0.014 | 0.447 | 0.139 | 0.581 | 0.786 | 0.13 | — |  |  |  |  |
| 16. Sad Accuracy | r | 0.04 | 0.07 | 0.10 | 0.01 | 0.05 | -0.01 | 0.02 | -0.12 | 0.07 | -0.1 | 0.51****** | 0.19 | 0.07 | 0.18 | -0.03 | — |  |  |  |
|  | *p* | 0.832 | 0.717 | 0.6 | 0.974 | 0.811 | 0.968 | 0.916 | 0.542 | 0.714 | 0.615 | 0.005 | 0.332 | 0.716 | 0.344 | 0.861 | — |  |  |  |
| 17. Surprise Accuracy | r | -0.22 | 0.22 | 0.22 | -0.24 | -0.19 | -0.20 | -0.30 | -0.33 | -0.08 | -0.12 | 0.22 | -0.02 | 0.05 | -0.09 | -0.08 | 0.02 | — |  |  |
|  | *p* | 0.25 | 0.26 | 0.252 | 0.206 | 0.321 | 0.289 | 0.117 | 0.079 | 0.668 | 0.553 | 0.247 | 0.93 | 0.794 | 0.652 | 0.681 | 0.906 | — |  |  |
| 18. Happiness versus Fear ERD (Beta) | r | -0.08 | -0.42***** | 0.04 | 0.12 | 0.08 | 0.20 | 0.25 | -0.01 | 0.01 | 0.05 | -0.08 | -0.04 | 0.12 | -0.01 | 0.11 | -0.06 | -0.14 | — |  |
|  | *p* | 0.663 | 0.02 | 0.817 | 0.538 | 0.676 | 0.299 | 0.183 | 0.976 | 0.978 | 0.784 | 0.666 | 0.83 | 0.546 | 0.945 | 0.569 | 0.74 | 0.458 | — |  |
| 19. Happiness versus Sadness ERD (Beta) | r | -0.40***** | -0.09 | 0 | -0.31 | -0.36 | -0.26 | -0.15 | -0.34 | -0.22 | < .001 | -0.07 | -0.30 | 0.20 | -0.01 | -0.01 | 0.16 | 0.21 | 0.47****** | — |
|  | *p* | 0.027 | 0.648 | 0.992 | 0.097 | 0.051 | 0.171 | 0.444 | 0.066 | 0.246 | 0.997 | 0.714 | 0.118 | 0.301 | 0.945 | 0.953 | 0.416 | 0.273 | 0.01 | — |

*Note.* ^1^ The highlighted grey areas represent Pearson’s r values and the unhighlighted areas represent Spearman’s Rho values.

^2^ The r value of either Pearson or Spearman is represented by rows with “r” and the *p* value is represented by rows with “*p*”.

^3^ * *p* < .05, ** *p* < .01 and ******* *p* < .001.
